# The behavioral and physiological effects of high‐fat diet and alcohol consumption: Sex differences in C57BL6/J mice

**DOI:** 10.1002/brb3.708

**Published:** 2017-04-20

**Authors:** Rachel R. Gelineau, Nicole L. Arruda, Jasmin A. Hicks, Isabella Monteiro De Pina, Aikaterini Hatzidis, Joseph A. Seggio

**Affiliations:** ^1^ Department of Biological SciencesBridgewater State UniversityBridgewaterMAUSA

**Keywords:** alcohol, anxiety, behavior, C57BL/6, high‐fat diet, locomotor, mouse, preference, sex difference, type 2 diabetes

## Abstract

**Background and Objective:**

Animal studies can be a great tool to investigate sex differences in a variety of different ways, including behavioral and physiological responses to drug treatments and different “lifestyle variables” such as diets. Consumption of both high‐fat diets and alcohol is known to affect anxiety behaviors and overall health. This project investigated how high‐fat diet and alcohol access and its combination affected the behavior and physiology of male and female C57BL/6J mice.

**Method:**

Mice were separated into three food groups: high‐fat diet, 10% fat diet, and regular chow, and each group was paired with either water or 10% alcohol. Behavioral assays included diet and alcohol preference, light‐dark box, open field, and feeding and drinking measurements. Physiological measures included glucose tolerance tests and measurement of brain‐derived neurotrophic factor, insulin, and leptin levels.

**Results:**

Females and males differed in the open field, as male mice decreased activity, while females increased activity when consuming high‐fat diet. While females consumed more ethanol than males, alcohol consumption was able to improve glucose tolerance and increase anxiety in both sexes. Lastly, females were more resistant to the physiological changes caused by high‐fat diet than males, as females consuming high‐fat diet exhibited decreased insulin secretion, less change to brain‐derived neurotrophic factor levels, and better glucose tolerance than males consuming high‐fat diet.

**Conclusion:**

These results suggest that the response to high‐fat diet and alcohol consumption is sex dependent and that males are more affected both behaviorally and physiologically by high‐fat diet compared to females.

## 1 Introduction

According to the Center of Disease Control and Prevention, there are approximately 22.0 million people in the United States diagnosed with diabetes, 95% of which have type 2 diabetes mellitus (T2DM) (Johnson et al., 2014). Noninsulin‐dependent T2DM is caused by both environmental and genetic factors and impairs an individual's ability to regulate blood glucose levels through the inability of insulin to perform properly, which leads to the development of insulin resistance (Leahy, 2005). One of the most common environmental factors that contributes to the increased risk of developing T2DM is consumption of a high‐fat and high‐sugar diet. These high‐fat diets (HFDs) also contribute to the development of obesity, which is often, but not always, associated with T2DM, and can lead to both insulin and leptin resistance.

Behavioral changes, including anxiety, have also been linked to both obesity and T2DM. For example, the likelihood of experiencing anxiety is greater than 50% in type 2 diabetics compared to the general population (Collins , Corcoran, & Perry, 2009) and also increased in individuals with obesity (Gariepy, Nitka, & Schmitz, 2010). In addition, children of both sexes with a high BMI were more likely to have a form of behavioral disorder (Rofey et al., 2009). While obesity and T2DM can lead to increased anxiety in humans, whether the increased anxiety is due to the poor diet directly or genetic predisposition to those conditions remains a bit unclear. Animal studies which investigate anxiety in genetically modified diabetic and obese models on normal diet show different outcomes depending upon the mutation. The monogenetic mutant *db/db* (diabetic) mice exhibit decreased anxiety‐like behaviors (Sharma, Elased, Garrett, & Lucot, 2010), but there is also evidence that the *ob/ob* (obese) mouse may show increased anxiety (Asakawa et al., 2003). On the other hand, animal studies have shown a relationship between anxiety‐like behaviors and HFD consumption as anxiety measures are increased in both male (Zemdegs et al., 2016) and female (Sivanathan, Thavartnam, Arif, Elegino, & McGowan, 2015) rodent models exposed to HFD. Saturated fats (i.e., the lard in many HFDs) might be the main culprit for these changes in behavior in rodent studies (Mizunoya et al., 2013). A human study also found a connection between HFD and increased anxiety as well (Bonnet et al., 2005).

Similar to HFD consumption, numerous studies have shown that high levels of alcohol consumption can increase anxiety levels in both humans (Kushner, Abrams, & Borchardt, 2000) and animal models (Gilpin, Karanikas, & Richardson, 2012; Popović, Caballero‐Bleda, Puelles, & Guerri, 2004). Alcoholics are also known to have higher levels of anxiety during drinking periods (Caldwell et al., 2002; Swendsen et al., 1998) and when experiencing withdrawal (Canan & Ataoglu, 2008), which are corroborated by animal studies (Doremus, Brunell, Varlinskaya, & Spear, 2003; Valdez et al., 2002). Additionally, individuals with high anxiety sensitivity are more likely to drink heavily than nonanxious populations (Stewart & Zeitlin, 1995). On the other hand, it seems that low‐to‐moderate alcohol consumption produces little‐to‐no changes in anxiety levels (Bellos et al., 2013), so the effects of alcohol being anxiogenic are limited to heavy consumption only.

In addition to modulating anxiety levels, alcohol consumption also affects leptin and insulin levels. Alcohol consumption produces reductions in circulating leptin (Röjdmark, Calissendorff, & Brismar, 2001) and blockade of leptin pathways leads to the cessation of drinking (Blednov, Walker, & Harris, 2004). In addition, moderate alcohol consumption can lead to reductions in insulin secretion and improvements in insulin resistance (Lazarus, Sparrow, & Weiss, 1997). These results illustrate a connection between alcohol consumption and T2DM. Both binge drinking and moderate consumption of alcohol can affect the progression and risk of T2DM, but they appear to have different influences on the disease. Excessive drinking can lead to an increased risk of developing T2DM in both men (Kao, Puddey, Boland, Watson, & Brancati, 2001) and women (Carlsson, Hammar, Grill, & Kaprio, 2003). Conversely, low‐to‐moderate alcohol consumption seems to produce a lower risk of developing T2DM (Baliunas et al., 2009; Koppes, Dekker, Hendriks, Bouter, & Heine, 2005). These results indicate a “J”‐shaped curve regarding the effects of alcohol consumption on T2DM risk as some alcohol consumption imparts a reduced risk compared to not drinking at all, but excessive drinking creates increased possibility of developing the disease (Carlsson, Hammar, & Grill, 2005).

While numerous studies have investigated the links between T2DM and alcohol consumption, few experiments have directly dealt with the interaction between HFD consumption and alcohol consumption in either humans or rodent models. This study aims to determine how combined HFD and alcohol consumption affects a variety of behaviors and overall health in both male and female C57BL/6J (B6) mice. The B6 mouse was used in this study because it becomes obese and type 2 diabetic‐like when given a HFD (Surwit, Kuhn, Cochrane, McCubbin, & Feinglos, 1988) and has one of the highest levels of alcohol consumption among all mouse strains (Yoneyama, Crabbe, Ford, Murillo, & Finn, 2008). The experimental procedures in this study aim to uncover sex differences for the following behavioral and physiological characteristics in response to HFD, moderate alcohol consumption, or both combined: diet and alcohol consumption patterns, anxiety‐like behaviors, T2DM‐like symptoms including glucose tolerance and hormonal changes, locomotor activity patterns, and diet and fluid preferences.

## 2 Materials and Methods

### 2.1 Statement on animal care

All animal studies were carried out with the approval from Bridgewater State University's Institutional Animal Care and Use Committee (IACUC).

### 2.2 Experiment 1: Physiological and behavioral effects of combined high‐fat diet and alcohol

#### 2.2.1 Animals

Forty‐eight male (M) and 48 female (F) C57BL/6J (B6) mice were purchased from Jackson Laboratories (Bar Harbor, ME, USA), approximately 6 weeks of age and upon their arrival, were housed individually and placed in a 12‐hr light:12‐hr dark (LD) cycle with a regular chow (RC, 3.36 kcals per gram with kcal percentages 13.4% fat, 29.8% protein, and 56.8% carbohydrate, LabDiet 5001, St. Louis, MO, USA). After a 4‐ to 5‐day acclimation, each mouse was given a diet of 60% high‐fat diet (HFD, 5.10 kcals per gram with kcal percentages 61.6% fat, 18.1% protein, and 20.3% carbohydrate, TestDiet 58Y1, St. Louis, MO, USA), 10% fat diet (TEN, 3.76 kcals per gram with kcal percentages 10.2% fat, 18.0% protein, and 71.8% carbohydrate, TestDiet 58Y2), or remained on the regular chow (RC), and were given a drink of either 10% alcohol (EtOH) or continued with water (H_2_O) (all in nonfree‐choice paradigms). This experiment utilized two control foods: RC, which is most commonly used as food for rodents in animal studies and TEN, which is ingredient‐matched HFD to TEN, where the only difference is the replacement of fat with carbohydrate. The two control diets were used as previous work from my laboratory (Hicks et al., 2016) has found that liquid consumption is reduced in mice consuming this version of HFD compared to RC and as this study also investigated alcohol consumption, the TEN diet was utilized to make that comparison controlling for ethanol consumption. This experiment had 12 groups each with a *N* = 8: 1) M/HFD/ H_2_O, 2) F/HFD/ H_2_O, 3) M/HFD/EtOH, 4) F/HFD/EtOH, 5) M/TEN/ H_2_O, 6) F/TEN/ H_2_O, 7) M/TEN/EtOH, 8) F/TEN/EtOH, 9) M/RC/EtOH, 10) F/RC/EtOH, 11) M/RC/ H_2_O, 12) F/RC/ H_2_O (*N* = 8). Weekly measurements of body weight, and food and fluid intake were measured and recorded. Food consumption was converted into kilocalories (kcals) per week consumed, while ethanol consumption was converted into grams per kilogram (g/kg).

#### 2.2.2 Assessment of explorative and anxiety‐like behaviors

At approximately 15–16 weeks of age, open field tests and light‐dark box tests were performed for all mice. In the open field tests, each mouse was individually placed into an open field arena within a SmartCage™ software system (AfaSci Inc., Redwood City, CA, USA) as previously described (Hicks et al., 2016). Each mouse was allotted 10 min in the cage and the infrared beams measured activity counts, activity time, distance, velocity, left and right rotations, and rears for all zones. Zone 5 was considered the center of the arena, and time spent there was also measured. All variables were analyzed for the first 5 min and the total time. For the light‐dark box tests, each mouse was individually placed in the light side and the mouse's movements were monitored for 10 min. Time spent in the dark side, number of entries into the dark compartment, and time to first entry into the dark zone were measured.

#### 2.2.3 BDNF

Brain‐derived neurotrophic factor (BDNF) has been implicated in anxiety and feeding behavior, and both alcohol and HFD are known to reduce its levels. Whole‐brain BDNF was measured for each individual mouse to determine if there are widespread alterations throughout the brain. After euthanasia, the entire brains of the mice minus the cerebellum were removed and immediately stored in −80°C. After storage, whole‐brain tissue homogenates were created in a cocktail containing Pierce IP Lysis buffer (Thermo Scientific, Rockford, IL, USA) and protease inhibitor (Halt Protease Inhibitor Single‐Use Cocktail EDTA‐Free 100×; Thermo Scientific) at a ratio of 100 μl of protease inhibitor for each 10 ml of lysis buffer, and 0.2 ml of protease/lysis cocktail was added for each 0.1 g of brain tissue. A low target concentration (working dilution 1:2) of sample and sample diluent buffer was created and then used in a BDNF ELISA (Mouse BDNF PicoKine ELISA Kit, Boster Biological Technology Co., Pleasanton, CA, USA). Males and female BDNF levels were separately normalized to their RC H_2_O control groups for analyses.

#### 2.2.4 Assessment of diabetic‐like phenotype

Glucose tolerance tests (GTTs) were performed on all mice at approximately 17 weeks of age. Following a 12‐hr fast, where food was removed and alcohol was replaced with water, a small prick was made at the tip of the mouse's tail and a baseline blood glucose level (Time 0) was determined using One‐Touch Ultra‐2 glucose monitors. An intraperitoneal injection of 2 g/kg glucose was given to each mouse and blood glucose levels were subsequently measured at 30, 60, and 120 min postinjection. Four‐hour fasting insulin and leptin levels were measured after 18 weeks of age. Blood was collected from each mouse and allowed to clot and was centrifuged at 4°C for 20 min at 2000 *g* in order to obtain serum. ELISAs for insulin (Ultra Sensitive Mouse Insulin ELISA Kit, Crystal Chem Inc., Downers Grove, IL, USA) and leptin (Mouse Leptin ELISA Kit; Crystal Chem) were conducted for both male and female mice.

#### 2.2.5 Statistical analysis

All tests and measurements were conducted during the light phase. Area under the curve (AUC) was calculated for each mouse for the GTTs. A two‐way ANOVA was used to assess differences for g/kg alcohol consumed. Body weight, fluid and food intake, kcals consumed, parameters in the behavioral assays, fasting glucose, AUC for GTTs, insulin, leptin, and BDNF levels were all analyzed using a three‐way ANOVA with Tukey HSD post hoc pairwise comparisons.

### 2.3 Experiment 2: Diet and ethanol preference and locomotor activity profile

#### 2.3.1 Animals

Thirty‐one male (M) and 22 female (F) B6 mice were purchased from Jackson Laboratories, approximately 6 weeks of age, housed individually, and, upon their arrival, were placed in a 12‐hr:12‐hr LD cycle with RC. Male and female mice were placed into either a food preference experiment (HFD and RC) or a drink preference experiment (10% EtOH and H_2_O). Two control groups of males (*N* = 6) and females (*N* = 5) consuming only RC and H_2_O were used to assess overall fluid and food consumption and activity profiles between sexes.

#### 2.3.2 Assessment of food preference

This experiment aimed to determine if the addition of a bottle of alcohol can alter HFD preference in terms of percentage of HFD out of total food intake. There were four experimental groups: 1) M/HFD+RC/EtOH (N = 7), 2) M/HFD+RC/ H_2_O (N = 6), 3) F/HFD+RC/EtOH (N = 4), 4) F/HFD+RC/ H_2_O (N = 4). Mice aged 8–12 weeks were given both a HFD and RC diet and a single bottle (forced) of H_2_O to drink, and preferences were recorded for weeks 10–12 before the switch. After Week 12, half of the food preference mice of each sex remained on H_2_O and half were given forced 10% EtOH for 3 weeks (weeks 13–15). Weekly measurements of food preference percentage, total grams of high‐fat diet consumed divided by total grams of food consumed, along with food and fluid intake were recorded.

#### 2.3.3 Assessment of drink preference

This experiment aimed to determine if the addition of HFD would alter the preference for alcohol in a two‐bottle choice paradigm. There were four experimental groups: 1) M/ H_2_O +EtOH/TEN (*N* = 6), 2) M/ H_2_O +EtOH/HFD (*N* = 6), 3) F/ H_2_O +EtOH/TEN (*N* = 5), 4) F/ H_2_O+EtOH/HFD (*N* = 5). Drink preference mice aged weeks 8–12 were given TEN and a two‐bottle choice of 10% EtOH and H_2_O, and preference was recorded for weeks 10–12 before the switch. After Week 12, half of the drink preference mice remained on the TEN and half were given forced HFD for 3 weeks (weeks 13–15). Weekly measurements of fluid preference, total EtOH consumed divided by total fluid consumed, and fluid and food intake were measured.

#### 2.3.4 Assessment of locomotor activity

Home‐cage locomotor activity was continuously recorded by IR beams located on the middle of each cage, above the cage lid (Starr Life Sciences, Oakmont, PA, USA) through the number of beam breaks. Total average locomotor activity, using Actiview (Starr Life Sciences) and a bout analysis ClockLab (Actimetrics, Wilmette, IL, USA), was calculated as previously described (Nascimento, Hicks, Carlson, Hatzidis, Amaral, Logan, et al., 2016; Nascimento, Hicks, Carlson, Hatzidis, Amaral, & Seggio, 2016) for both before (weeks 10–12) and after (weeks 13–15) the diet/drink switch.

#### 2.3.5 Statistical analysis

Independent t‐tests were used to determine sex differences in locomotor activity, and food and fluid consumption between the two control groups. Paired *t*‐tests were used to assess before versus after activity parameters within each group. For the experimental groups, two‐way ANOVAs were conducted with Tukey HSD post hoc pairwise comparisons using fluid preference, food preference, food and fluid intake, and locomotor activity parameters, to determine sex differences.

## 3 Results

### 3.1 Experiment 1

#### 3.1.1 Body mass

**Figure 1:**
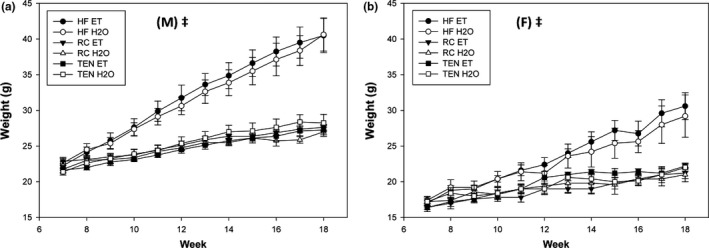
Body Weight. (a) Body weight (g) males and (b) body weight females. Throughout the entire study, female mice weighed less than males. As expected, HFD access led to weight gain compared to RC and TEN in both male and female mice. Additionally, male mice were more susceptible to weight gain and gained more weight on HFD than females. Circles indicate HFD, triangles indicate RC, and squares indicate TEN. Open circles indicate water, and filled circles indicate alcohol. (M) and (F) refer to male and female graphs, respectively. ‡ significant sex difference at *p *<* *.05

Initial body weights (Week 7, prior to treatments) were significantly different between males and females (*F*_1,82_ = 443.02, *p *<* *.001), but did not differ among the diet (*F*_2,82_ = 1.21, *p *=* *.30) or alcohol groups (*F*_1,82_ = 0.28, *p *=* *.60). From Week 8 until Week 18, there were both sex and diet effects on body weight, where female mice were significantly lighter than males and all mice consuming HFD were significantly heavier than mice consuming TEN and RC (all *p *<* *.05). In addition, a sex by diet interaction was uncovered for total weight gain (Week 18 weight minus Week 8 weight) over the course of the experiment (*F*_2,82_ = 3.99, *p *=* *.023). Whereas males and females consuming RC and TEN gained similar amounts of weight during the experiment (*p *=* *.99, .98, respectively), females consuming HFD gained less weight than males (*p *=* *.002) (Figure 1a,b).

#### 3.1.2 Food consumption

**Figure 2:**
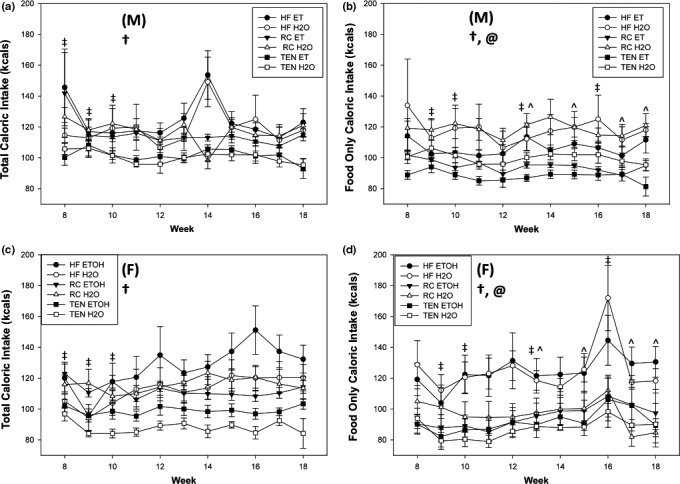
Food Consumption. (a) kcals per week with EtOH males, (b) kcals with EtOH females, (c) kcals per week without EtOH males, and (d) kcals without EtOH females. Diet significantly affected both total kcals per week (food plus ethanol) and kcals without ethanol (food only), as HFD and RC mice exhibited increased kcals than TEN (except Week 14, where HFD was greater than the other groups). For many weeks during the experiment, food consumption was reduced in female mice compared to males. Lastly, weeks 13, 15, 17–18, mice consuming both HFD and ethanol consumed more kcals than RC ethanol, but HFD water and RC water were equal. Ethanol consumption reduced food intake in both sexes. Open circles indicate water, and filled circles indicate alcohol. (M) and (F) refer to male and female graphs, respectively. ^ significant diet x drink interaction, @ indicates ethanol difference, † significant diet difference, ‡ significant sex difference at *p *<* *.05

Initially, there was a sex difference in total kcals per week (diet plus ethanol) from weeks 8 to 10 where females ingested less kcals than males (all *p *<* *.05), but for the remainder of the experiment (except for Week 14), males and females showed equal total kcal consumption. Additionally, diet significantly influenced kcals ingested where HFD consumption and RC consumption were equal and significantly greater than TEN (all *p *<* *.05), except for Week 14, where HFD was significantly greater than RC and TEN (*p *<* *.05) (Figure 2a,b). If kcals from food only was analyzed (i.e., no ethanol kcals), animals consuming ethanol exhibited reduced kcals from food compared to water animals, regardless of sex or diet throughout the experiment (all *p *<* *.05). Once again, animals consuming HFD and RC exhibited increased food consumption compared to TEN (all *p *<* *.05) throughout the experiment. Females consumed less food than males for weeks 9–10, 13, and 15 (all *p *<* *.05), but no other weeks (all *p *>* *.10). For weeks 13, 15, and 17–18, a diet by fluid interaction revealed that animals consuming HFD and ethanol consumed more food calories than RC ethanol (all *p *<* *.05), but their water counterparts exhibited equal food consumption (all *p *>* *.10). This result means that toward the end of the experiment, mice consuming HFD did not reduce their food intake when consuming ethanol, as the RC mice did (Figure 2c,d).

#### 3.1.3 Alcohol consumption

**Figure 3:**
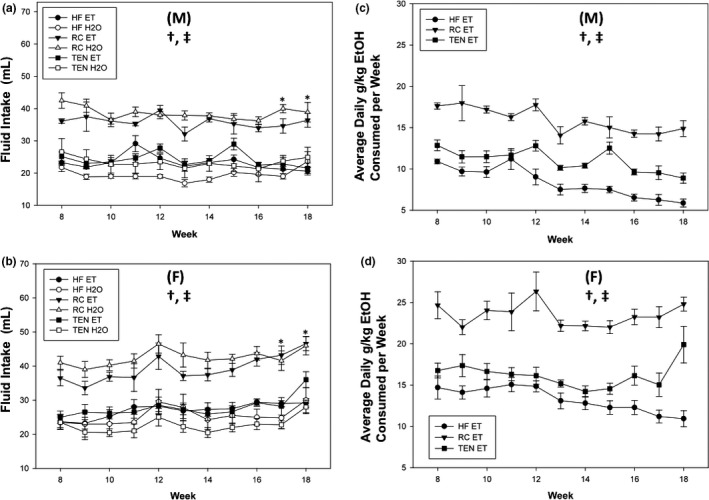
Alcohol consumption. (a) Alcohol consumed males (mls), (b) alcohol consumed females, (c) g/kg ethanol males, and (d) g/kg ethanol females. Female mice consumed more ethanol in terms of volume and g/kg than male mice. Mice consuming RC exhibited increased g/kg dose of ethanol compared to TEN, which in turn was significantly more than HFD. Circles indicate HFD, triangles indicate RC, and squares indicate TEN. Open circles indicate water, and filled circles indicate alcohol. (M) and (F) refer to male and female graphs, respectively. * significant pairwise difference between male and female ethanol consumption, † significant diet difference, ‡ significant sex difference at *p *<* *.05

Throughout the experiment, overall consumption of ethanol and water was reduced in mice consuming TEN and HFD compared to controls (all *p *<* *.05). From Week 12 until Week 18, female mice consumed more ethanol compared to male mice (all *p *<* *.05). Sex by fluid interactions were found for Week 17 (*F*_1,82_ = 4.36, *p *=* *.041) and Week 18 (*F*_1,82_ = 6.96, *p *=* *.012). While male and female mice drinking water exhibited similar consumption levels for the last two weeks (*p *=* *.38, .073), females had higher ethanol consumption than their male counterparts (both <0.001) (Figure 3a,b). Lastly, sex and diet differences were found in grams per kilograms ethanol consumption throughout the experiment (except Week 11), where females consumed higher doses than males, and mice consuming RC consumed more ethanol than HFD or TEN, but TEN consumed more per body mass than HFD (all *p *<* *.05) (Figure 3c,d).

#### 3.1.4 Open field

**Figure 4:**
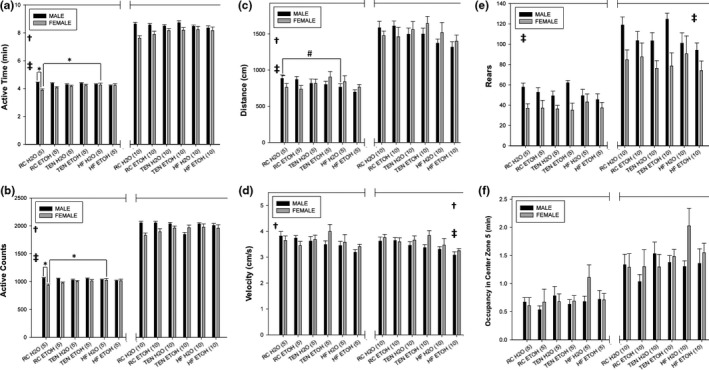
Open field. (a) Active time, (b) active counts, (c) distance traveled, (d) velocity, (e) rears, and (f) Zone 5 (center zone) time. For the first 5 min, mice consuming HFD exhibited reduced activity time and counts, distance traveled, and velocity compared to mice consuming TEN or RC. Females consuming water and HFD exhibited increased active counts and time compared to water/RC females and this difference was not seen in males. Additionally, water/RC males moved a greater distance than water/HF males. Female mice overall moved more in the open field as indicated by increased active time and counts, distance, and velocity and also exhibited increased rears. For the total 10 min, sex differences still existed for velocity and rearing, but the differences in the other variables were no longer present. Alcohol produced no alterations to open field behaviors. Center zone time was unaffected by any variable. Icons and bars on the left side of the break indicate data and significant differences from the first 5 min, while the icons and bars on the right are for the total 10‐min assay. Black bars indicate males, and gray bars indicate females. * significant pairwise difference, † significant diet difference, ‡ significant sex difference at *p *<* *.05; # pairwise difference at *p* = .054. Icons located on the left side indicate significant differences for the first 5 min, while icons on the right side indicate differences for the total 10‐min assay

The data summarized here are for the open field during the first 5 min. A sex by diet interaction was uncovered for both active time (*F*_2,82_ = 7.31, *p *=* *.001) and active counts (*F*_2,82_ = 7.42, *p *=* *.001). Female mice consuming HFD displayed increased active time (*p *=* *.011) and counts (*p *=* *.010) compared to RC, but no differences were found between HFD and TEN (*p *=* *.078, .073 for time and counts, respectively). Female mice consuming RC exhibited significantly lower active time and counts (both *p *<* *.001) compared to RC males, but exhibited no differences when consuming HFD (both *p *=* *.99) or TEN (*p *=* *.51, .49) (Figure 4a,b). A diet by sex interaction was also uncovered for distance (*F*_2,82_ = 4.58, *p *=* *.013) (Figure 4c); subsequent pairwise comparisons showed that male mice consuming HFD moved less distance compared to RC (*p *=* *.054), but this difference was not seen in female mice (*p *=* *.91). Diet significantly affected velocity in the open field (*F*_2,82_ = 3.63, *p *=* *.031); mice consuming HFD moved significantly slower than TEN (*p *=* *.040) but not slower than RC (*p *= .084) (Figure 4d). Female mice reared less than male mice across all groups (*F*_1,82_ = 26.04, *p *<* *.001) (Figure 4e). Diet (*F*_2,82_ = 1.95, *p *=* *.15) nor sex (*F*_1,82_ = 0.97, *p *=* *.33) had any effect on Zone 5 (center) time (Figure 4f). Lastly, ethanol consumption produced no alterations to open field behaviors for any of the six parameters (all *p *> .10).

For the total 10 min of the open field, the differences found during the first 5 min for active time and count, and distance, disappear (all *p *> .10). However, sex of the animals and diet still affected velocity during the full 10‐min test, where females moved at a higher velocity than males (*F*_1,82_ = 5.34, *p *=* *.023), and mice consuming HFD (*F*_2,82_ = 7.65, *p *=* *.001) moved slower than both RC (*p *=* *.001) and TEN (*p *=* *.013). Similar to what was found during the first 5 min, females reared less than males for the full 10‐min duration (*F*_1,82_ = 14.22, *p *<* *.001). Lastly, no differences were found for any variable for Zone 5 time (all *p *>* *.08). Again, ethanol consumption produced no alterations to open field behaviors (all *p *>* *.10).

#### 3.1.5 Light‐dark box

**Figure 5:**
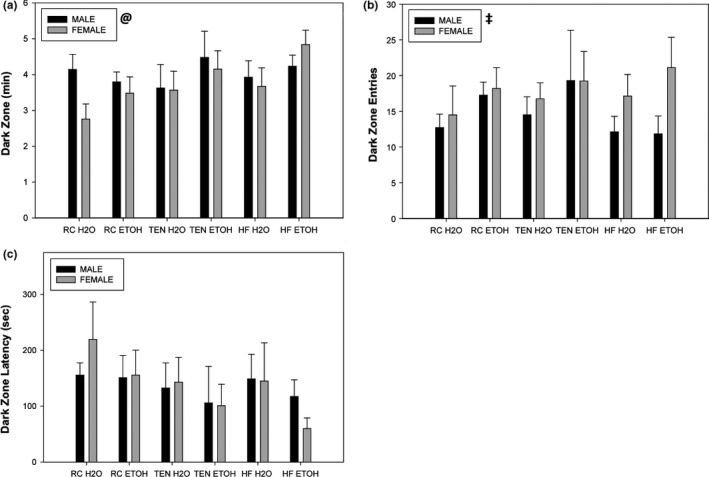
Light:dark Box. (a) Dark zone time, (b) dark zone entries, and (c) dark zone latency. Alcohol consumption regardless of sex or diet produced increases in dark zone time. Female mice exhibited increased entries and transitions compared to male mice, while no variable affected dark zone latency. Black bars indicate males, and gray bars indicate females. @ significant alcohol difference, ‡ significant sex difference at *p *<* *.05

Ethanol consumption led to more time spent in the dark side of the light‐dark Box, indicating increased anxiety‐like behavior (*F*_1,76_ = 4.13, *p* = .046) (Figure 5a). Diet (*F*_2,76_ = 1.78, *p* = .18) nor sex (*F*_1,76_ = 1.17, *p* = .28) had any influence on time in the dark side. Females had more dark entries and transitions compared to males (*F*_1,73_ = 4.43, *p* = .039), but diet (*F*_2,73_ = 0.33, *p* = .73) nor alcohol consumption (*F*_1,73_ = 2.26, *p* = .14) had any effect (Figure 5b). Dark zone latency (i.e., time to the first dark zone entry) was unaffected by any independent variable (all *p *>* *.09) (Figure 5c).

#### 3.1.6 BDNF

**Figure 6:**
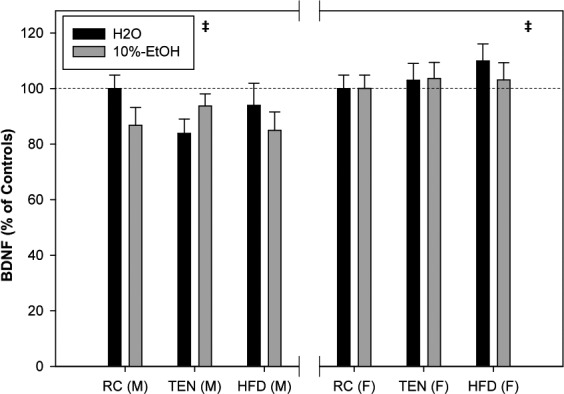
Whole‐brain BDNF. When normalized to their respective sex controls, males overall exhibited small but significant decreases to BDNF compared to females. Black bars indicate water, and gray bars indicate 10% EtOH. The dashed line indicates 100% RC H_2_O expression. ‡ significant sex difference at *p *<* *.05

In order to control for the variability between ELISA plates, both male and female whole‐brain BDNF levels were normalized to a percentage of RC H2O for each sex to 100%. Normalized BDNF levels were significantly lower in male mice compared to females accounting for all treatments (*F*_2,82_ = 15.93, *p *<* *.001). Neither diet (*F*_2,82_ = 0.13, *p* = .88) nor ethanol (*F*_1,82_ = 0.92, *p* = .34) produced significant changes to BDNF (Figure 6).

#### 3.1.7 Glucose tolerance, insulin, and leptin

**Figure 7:**
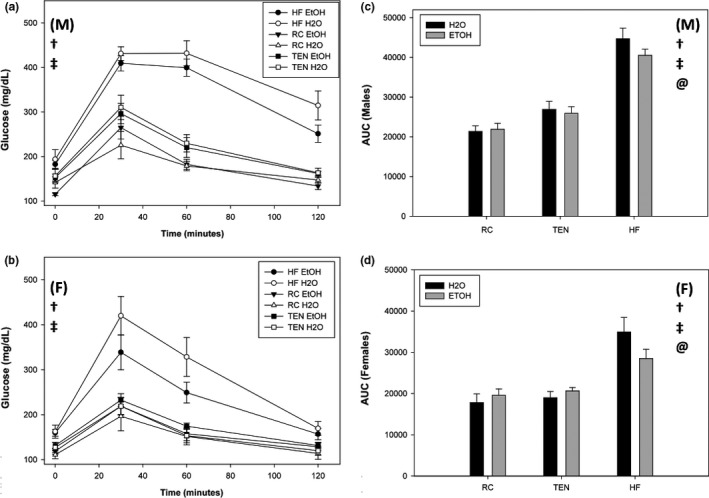
Glucose tolerance. (a) Glucose over time males, (b) glucose over time females, (c) area under the curve males, and (d) area under the curve females. When consuming HFD, males exhibited worse fasting glucose and glucose tolerance than females. Additionally, mice consuming both HFD and ethanol had improved glucose tolerance than mice consuming HFD and water regardless of sex, but ethanol had no effect on mice consuming RC or TEN. Circles indicate HFD, triangles indicate RC, and squares indicate TEN. Open circles indicate water, and filled circles indicate alcohol. Black bars indicate males, and gray bars indicate females. (M) and (F) refer to male and female graphs, respectively. † significant diet difference, @ significant alcohol difference, ‡ significant sex difference, at *p *<* *.05

Females exhibited reduced fasting blood glucose levels (Time 0) compared to males (*F*_1,80_ = 8.90, *p* = .004). Additionally, diet influenced fasting glucose levels (*F*_1,80_ = 29.48, *p *<* *.001), where animals consuming HFD displayed elevated fasting glucose compared to TEN (*p *<* *.001) and RC (*p *<* *.001) (Figure 7a,b). Alcohol produced no change to fasting glucose; interestingly, mice consuming TEN had higher fasting glucose than RC (*p* = .004). For glucose tolerance, AUC analysis uncovered a sex by diet interaction (*F*_2,78_ = 4.72, *p* = .011) and a diet by fluid interaction (*F*_2,78_ = 3.66, *p* = .030). Whereas male and female mice consuming RC exhibited similar glucose tolerance (*p* = .53), male mice consuming HFD displayed poorer glucose tolerance than HFD females (*p *<* *.001); in addition, male mice consuming TEN also displayed poorer glucose tolerance than females eating the TEN diet (*p* = .009). Ethanol consumption significantly improved glucose tolerance in mice consuming HFD (*p* = .050), but had no effect in animals consuming RC (*p* = .99) or TEN (*p* = .99) diets (Figure 7c,d).

**Figure 8:**
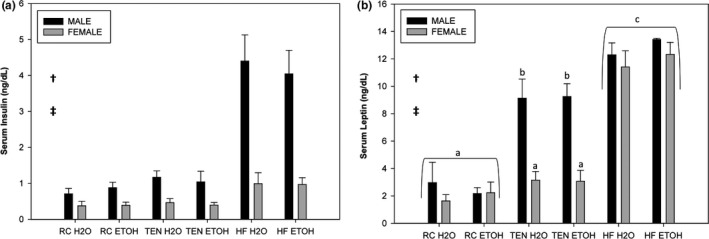
Serum insulin and leptin. (a) Insulin and (b) leptin. All mice consuming HFD exhibited increased insulin and leptin compared to mice consuming TEN and RC. Female mice overall had lower levels of insulin and leptin compared to males. Lastly, male mice consuming TEN had elevated levels of leptin compared to female TEN mice. Black bars indicate males, and gray bars indicate females. † significant diet difference, ‡ significant sex difference, and different letters indicate significantly different at *p *<* *.05

As expected, insulin was elevated in all mice consuming HFD (*F*_2,78_ = 31.11, *p *<* *.001) compared to RC and TEN (both *p *<* *.001). Additionally, female mice overall exhibited lower insulin levels compared to males (*F*_1,78_ = 46.73, *p *<* *.001) (Figure 8a). Ethanol produced no alterations to insulin (*F*_2,78_ = 0.033, *p* = .86). A sex by diet interaction was uncovered for leptin levels (*F*_2,82_ = 12.96, *p *<* *.001). Both male and female mice consuming HFD exhibited increased leptin levels compared to RC (both *p* = .001 for males and females) and TEN (both *p* = .001). Interestingly, male mice on TEN exhibited elevated levels of leptin compared to RC mice (*p *<* *.001), but it was still lower than mice consuming HFD (*p* = .001); this was not found for females (*p* = .72). Mice of the same sex had similar leptin levels for HFD (*p* = .86) and RC (*p *=* *.98), but not TEN (*p* = .001) (Figure 8b). Ethanol consumption had no effect on leptin levels (*F*_2,82_ = 0.41, *p* = .52).

### 3.2 Experiment 2

#### 3.2.1 Diet preference

**Figure 9:**
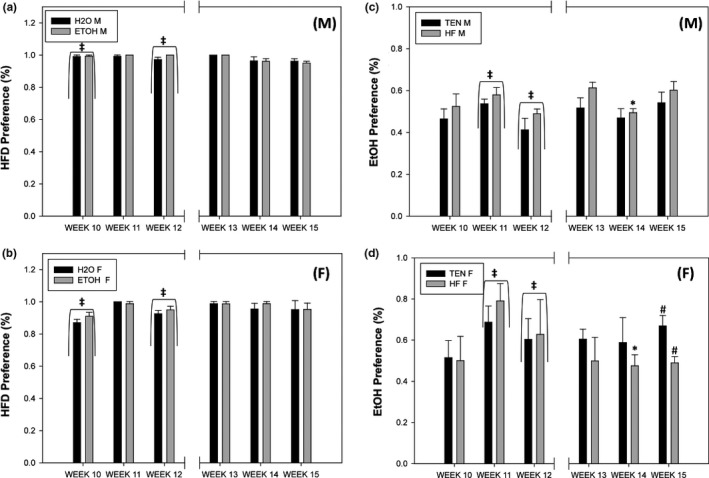
Diet and alcohol preferences. (a) HFD preference males, (b) HFD preference females, (c) ethanol preference males, and (d) ethanol preference females. Initially, females exhibited reduced HFD preference compared to males, but females increased their preference over time. Alcohol produced no effect on HFD preference. Alcohol preference was elevated in female mice compared to male mice, but male and female preference equalized as the experiment continued. The addition of HFD produced a significant reduction in alcohol preference for females during Week 14 and a moderate reduction in Week 15; males were unaffected by HFD appearance. Left side of the graph indicates before the addition of the new diet or liquid (weeks 10–12), while the right side (weeks 13–15) indicates the preferences after. (M) and (F) refer to male and female graphs, respectively. * significant pairwise difference, ‡ significant sex difference at *p *<* *.05; # pairwise difference at *p* = .069

For weeks 10 (*F*_1,17_ = 63.68, *p *<* *.001) and 12 (*F*_1,17_ = 14.08, *p* = .002), but not 11, female mice exhibited reduced HFD preference than males (Figure 9a,b). No differences were found in baseline HFD preference levels. After the addition of alcohol for weeks 13–15, the sex differences found earlier are no longer present (all *p *>* *.10), but this effect is more likely due to female mice increasing their food preference equal to males overall (i.e., the nonalcohol drinking female animals were equal to males and increased compared to earlier), rather than the addition of alcohol.

#### 3.2.2 Fluid preference

Initially, females exhibited increased ethanol preference compared to males during Week 11 (*F*_1,18_ = 12.45, *p* = .002) and Week 12 (*F*_1,17_ = 12.06, *p* = .003). No differences between the group to be switched and the control group were found for weeks 10–12 (all *p *>* *.10). After the switch in diet, a sex by treatment interaction was uncovered where female mice consuming HFD exhibited significantly reduced ethanol preference compared to males during Week 14 (*F*_1,17_ = 6.69, *p* = .019); additionally, HFD female preference for alcohol was moderately reduced during Week 15 (*F*_1,16_ = 7.63, *p* = .014; *p* = .069) compared to TEN consuming females. Over time, female preference for ethanol reduced during weeks 13–15 than weeks 10–12 (Figure 9c,d).

#### 3.2.3 Locomotor activity

**Figure 10:**
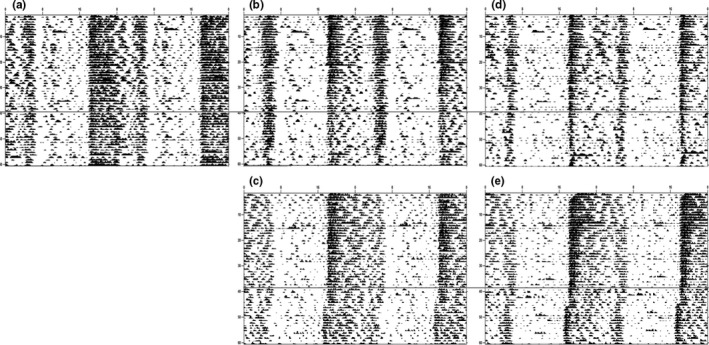
Male Actograms. (a) RC H_2_O (b) HFD/RC H_2_O (c) HFD/RC 10% EtOH (d) TEN H_2_O/10% EtOH (e) HFD H_2_O/10% EtOH. Representative actograms for each of the five treatment groups. The dashed line indicates when the food or drink switch occurred for the before and after analysis

**Figure 11:**
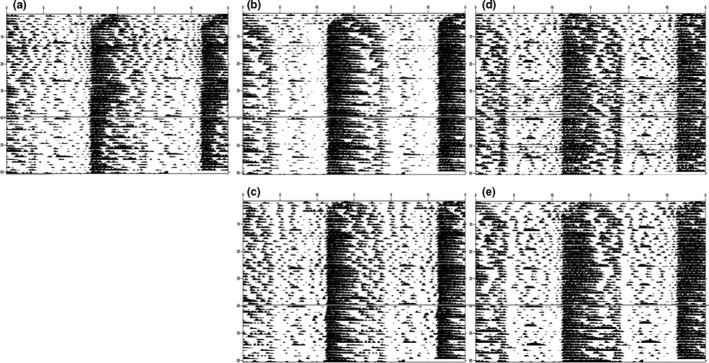
Female Actograms. (a) RC H_2_O (b) HFD/RC H_2_O (c) HFD/RC 10% EtOH (d) TEN H_2_O/10% EtOH (e) HFD H_2_O/10% EtOH. Representative actograms for each of the five treatment groups. The dashed line indicates when the food or drink switch occurred for the before and after analysis

The summary of locomotor behavior and graphical examples activity levels is summarized in Table 1 and Figures 10 and 11, respectively. Overall, independent t‐tests showed that female mice (throughout all three comparisons, controls, diet preference, and fluid preference) exhibited increased locomotor activity than males for all variables during the initial three‐week period (weeks 10–12, prior to the switching of fluid or diet) (for the controls, all *p *<* *.05), with the exception of light‐to‐dark (LD) ratio and bouts per day for the diet choice. Afterward (weeks 11–13), female mice still exhibited increased activity compared to males in the same categories as previously described (all *p *<* *.05), but this time, bouts per day were also different between males and females for the diet preference experiment (all *p *<* *.05). Switching either the diet or the fluid made no difference to locomotor activity during any of the experiments. Lastly, male mice fed HFD during the diet preference experiment exhibited decreased locomotor activity in terms of total, light, and dark activity, as well as counts per bout (all *p *<* *.05), compared to control males, regardless of ethanol access, for both before and after the alcohol switch; bouts per day and bout length were unaffected by diet (both *p *>* *.10). Before and after paired t‐tests revealed that controls of both sexes were not significantly different as time progressed (all *p *>* *.10). During the drink preference assay, male mice consuming HFD and free‐choice alcohol had reduced dark activity (*t*_1,5_ = 5.64, *p* = .003) and counts per bout (*t*_1,5_ = 2.84, *p* = .036) during weeks 13–15 (after) compared to weeks 10–12 (before), which was not the case for female mice (all *p *>* *.10). In addition, during the diet preference assay, male mice consuming free‐choice HFD exhibited reduced overall activity (*t*_1,5_ = 3.76, *p* = .013), dark activity (*t*_1,5_ = 3.48, *p* = .018), bout length (*t*_1,5_ = 3.07, *p* = .028), and counts per bout (*t*_1,5_ = 3.91, *p* = .011) for before vs. after, which once again was not found in female mice (all *p *>* *.09).

**Table 1:** Locomotor activity and bout analysis. Male mice exhibited decreased locomotor activity compared to females on RC. Additionally, males were also more sensitive to the negative effects of HFD exposure as they exhibited reduced activity over time compared to females. Alcohol produced no alterations to locomotor activity in either sex. *Italics* indicate significant differences between Before and After activity parameters, and different letters indicate significantly different from each other, at *p* <.05

Group	*N*	Sex	Diet	Fluid	Average	Light average	Dark average	LD Ratio	Bout length (min)	Counts per bout	Bouts per day
Before weeks 10–12											
Controls	5	M	RC	H_2_O	19.2 ± 1.7^a^	8.5 ± 0.6^a^	29.9 ± 3.1^a^	0.28 ± 0.09	47.8 ± 12.5^a^	226.3 ± 33^a^	11.1 ± 0.5^a^
	4	F	RC	H_2_O	44.5 ± 6.3^b^	17.2 ± 2.0^b^	71.8 ± 14.4^b^	0.29 ± 0.02	73.6 ± 3.9^b^	779.9 ± 220.0^b^	8.1 ± 1.1^b^
Diet Pref.	6	M	HFD/RC	H_2_O	*13.8 ± 1.1* ^*a*^ *	6.4 ± 0.8^a^*	*21.2 ± 2.7* ^*a*^ *	0.31 ± 0.02	*48.2 ± 5.0* ^*a*^	*226.3 ± 33.7* ^*a*^	8.1 ± 0.4*
	5	M	HFD/RC	10% EtOH	*14.4 ± 1.1* ^*a*^ *	5.38 ± 0.5^a^*	*23.46 ± 2.1* ^*a*^	0.23 ± 0.03	*48.4 ± 2.4* ^*a*^	*237.6 ± 18.7* ^*a*^	7.9 ± 0.4*
	4	F	HFD/RC	H_2_O	44.0 ± 7.0^b^	17.1 ± 3.0^b^	70.9 ± 15.5^b^	0.26 ± 0.07	76.0 ± 15.5^b^	787.5 ± 271.4^b^	7.8 ± 1.2
	4	F	HFD/RC	10% EtOH	39.3 ± 6.8^b^	14.5 ± 2.5^b^	64.2 ± 11.9^b^	0.23 ± 0.04	75.7 ± 9.5^b^	713.4 ± 168.2^b^	7.2 ± 0.3
Fluid Pref.	6	M	TEN	H_2_O/10% EtOH	20.1 ± 2.6^a^	8.0 ± 1.1^a^	32.1 ± 4.3^a^	0.25 ± 0.02	39.4 ± 2.8^a^*	231.3 ± 34.5^a^	11.4 ± 0.3^a^
	6	M	HFD	H_2_O/10% EtOH	20.9 ± 1.5^a^	8.5 ± 1.0^a^	*33.3 ± 2.1* ^*a*^	0.26 ± 0.02	36.3 ± 1.0^a^*	214.2 ± 14.7^a^	12.8 ± 0.4^a^
	4	F	TEN	H_2_O/10% EtOH	53.5 ± 12.3^b^	21.7 ± 9.7^b^	85.3 ± 15.1^b^	0.24 ± 0.06	73.6 ± 10.2^b^	847.1 ± 215.4^b^	8.1 ± 0.2^b^
	4	F	HFD	H_2_O/10% EtOH	48.5 ± 6.4^b^	15.9 ± 3.5^b^	81.2 ± 11.7^b^	0.20 ± 0.10	77.9 ± 15.3^b^	868.2 ± 181.9^b^	7.4 ± 0.8^b^
After weeks 13–15											
Controls	5	M	RC	H_2_O	17.6 ± 1.9^a^	7.7 ± 0.5^a^	27.6 ± 4.0^a^	0.29 ± 0.03	44.6 ± 3.8^a^	214.2 ± 31.1^a^	10.7 ± 0.2^a^
	4	F	RC	H_2_O	38.1 ± 6.5^b^	14.8 ± 1.2^b^	61.4 ± 14.3^b^	0.30 ± 0.10	61.1 ± 9.4^b^	580.0 ± 166.9^b^	8.9 ± 1.2^b^
Diet Pref.	6	M	HFD/RC	H_2_O	*11.9 ± 1.2* ^*a*^ *	5.3 ± 0.6^a^*	*18.5 ± 1.9* ^*a*^ *	0.29 ± 0.02	*41.1 ± 2.6* ^*a*^	*169.1 ± 18.6* ^*a*^	9.1 ± 0.4^a^*
	5	M	HFD/RC	10% EtOH	*12.1 ± 0.4* ^*a*^ *	5.0 ± 0.4^a^*	*19.2 ± 0.6* ^*a*^ *	0.26 ± 0.02	*41.6 ± 1.5* ^*a*^	*181.4 ± 9.9* ^*a*^	8.6 ± 0.5^a^*
	4	F	HFD/RC	H_2_O	37.0 ± 9.5^b^	15.2 ± 2.4^b^	58.7 ± 19.9^b^	0.30 ± 0.10	78.7 ± 23.9^b^	730.3 ± 353.7^b^	7.3 ± 1.3^b^
	4	F	HFD/RC	10% EtOH	35.3 ± 6.6^b^	12.2 ± 1.8^b^	58.3 ± 11.7^b^	0.21 ± 0.03	69.6 ± 7.3^b^	610.1 ± 150.9^b^	7.6 ± 0.2^b^
Fluid Pref.	6	M	TEN	H_2_O/10% EtOH	19.2 ± 2.5a	10.0 ± 1.4^a^	28.3 ± 3.7^a^	0.36 ± 0.02	34.4 ± 2.6^a^*	193.7 ± 32.0^a^	13.1 ± 0.5^a^*
	6	M	HFD	H_2_O/10% EtOH	18.0 ± 0.9a	8.9 ± 0.3^a^	*27.1 ± 1.4* ^*a*^	0.33 ± 0.01	34.6 ± 1.2^a^*	185.4 ± 11.5^a^	12.7 ± 0.4^a^*
	4	F	TEN	H_2_O/10% EtOH	52.5 ± 9.7b	19.1 ± 7.2^b^	85.8 ± 12.1^b^	0.21 ± 0.04	75.2 ± 5.7^b^	859.9 ± 152.9^b^	7.7 ± 0.5^b^
	4	F	HFD	H_2_O/10% EtOH	46.6 ± 4.8b	14.2 ± 2.0^b^	79.0 ± 8.4^b^	0.18 ± 0.02	95.4 ± 19.7^b^	1046.0 ± 291.5^b^	6.2 ± 0.9^b^

## 4 Discussion

This study reports numerous sex differences but also some similarities between male and female B6 mice in their response to HFD and alcohol exposure. Despite their smaller stature, the female mice in the current experiment consumed significantly more alcohol in terms of preference, and both volume and g/kg measures compared to males, similar to what was historically found in this mouse strain (Middaugh & Kelley, 1999; Yoneyama et al., 2008). While there were some instances of interactions between HFD and ethanol when both were consumed simultaneously for both sexes (e.g., glucose tolerance, food consumption, see below), only one of those diet by fluid interactions manifested in a sex difference—ethanol preference. Female mice when newly exposed to HFD reduced their ethanol preference ratio compared to males on HFD and females consuming TEN. These sex differences in alcohol drinking patterns may be explained by differences in peripheral gonadal hormones, ethanol metabolism, and neural mechanisms. Interestingly, male mice with the deleted *sry* gene and made gonadal female exhibit increased ethanol consumption compared to control males and similar to control females; females made gonadal male show the opposite effect and reduce their alcohol intake to levels of control males, indicating a possible role of estrogens in alcohol intake (Barker, Torregrossa, Arnold, & Taylor, 2010). In addition, female mice (Kishimoto et al., 2002) and humans (Cederbaum, 1999) have higher rates of ethanol metabolism and clearance compared to males. Lastly, female animals can recover faster from alcohol withdrawal perhaps due to sex differences in basal GABA (Devaud, Alele, & Ritu, 2003) or opioid signaling (Becker, Perry, & Westenbroek, 2012), indicating that males may experience more negative effects of ethanol consumption and withdrawal than females. Still, there are some similarities in their response to alcohol drinking as it increased anxiety as measured by the light‐dark box and improved glucose tolerance for HFD‐consuming mice regardless of sex.

In some instances, the sex differences were manifested by one sex being more resistant to behavioral or physiological changes brought on by a specific experimental treatment. One such example is female mice being more resistant to the obesogenic and T2DM‐generating effects of HFD exposure, where females exhibited reduced weight gain, hyperinsulinemia, and improved glucose tolerance compared to males. Some of these differences may be explained by differences in food consumption patterns; even though female mice consumed similar kcals as males from Week 11 onward, male mice did consume more calories due to food intake, which in turn means that males eating HFD did consume more levels of dietary fat compared to females. Nevertheless, these differences may persist even if weight gain is equal between the two sexes as female B6 mice which are similar in body weight to their male counterparts still can exhibit reduced hyperinsulinemia and glucose intolerance compared to males (Pettersson, Waldén, Carlsson, Jansson, & Phillipson, 2012). These sex differences in T2DM‐like symptoms and obesity might be due to differences between the gonadal hormones’ (estrogens and androgens) effects on metabolism. Treatment with estrogens or estradiol can reverse or prevent obesity, glucose intolerance, and insulin resistance in ovariectomized mice (Riant et al., 2009) and female ob/ob mice (Lundholm et al., 2008). Human studies have shown that postmenopausal women are more susceptible to develop T2DM and obesity compared to nonmenopausal women and are similar to men (as reviewed by Shi & Clegg, 2009). On the other hand, while androgens, including testosterone, can improve insulin sensitivity in males, neither castration (Macotela, Boucher, Tran, & Kahn, 2009) nor depletion of androgens (Varlamov et al., 2012; Yu et al., 2008) leads to obesity or T2DM‐like symptoms in animal models. In addition, HFD intake can lead to the decrease of testosterone (Cano et al., 2008) which can promote obesity and T2DM in males (Seidell, Björntorp, Sjöström, Kvist, & Sannerstedt, 1990), but to increases to estrogens in females (Shinoda, Latour, & Lavoie, 2002), which could be a protective mechanism (Fuente‐Martín, Argente‐Arizón, Ros, Argente, & Chowen, 2013). These studies suggest that reproductively capable females are more resistant to the negative physiological effects of HFD consumption by the actions of estrogens.

Another case where female animals may be more resistant than males is in alterations to leptin levels by specific types of diets. Surprisingly, male mice consuming the TEN diet exhibited significantly increased leptin compared to RC, but reduced compared to HFD. The TEN diet is an iso‐ingredient diet to HFD, and while having a reduced fat content, it has more sucrose (33% of the diet is sucrose as it is the replacement for the fat in the HFD) so it may be considered a high‐sugar diet compared to RC. A previous study found that high‐sugar diets can also produce hyperleptinemia (an indicator of leptin resistance) in male B6 mice compared to controls, but the sugar‐induced increases in leptin are not as elevated as in HFD (Sumiyoshi, Sakanaka, & Kimura, 2006). Indeed, diets high in fructose, one‐half of the disaccharide sucrose, can lead to leptin resistance by itself (Shapiro et al., 2008), which may be prevented and reversed in male rats if the sugar is removed (Shapiro, Tümer, Gao, Cheng, & Scarpace, 2011). Oppositely, female mice consuming TEN had no such increases to leptin levels. This dissimilarity in leptin levels due to sugar content is likely due to sex differences in lipogenesis from sugar sources. Female humans have lower plasma triglyceride levels in response to high carbohydrate diets and female mice have lower liver triglycerides as well, and incorporate less U‐^14^C‐glucose into liver triglycerides compared to male mice (Sheorain, Mattock, & Subrahmanyam, 1979). As increased levels of plasma triglycerides is an indicator of leptin resistance (Banks et al., 2004), it would appear that males are more likely to develop obesity and leptin resistance due to high‐sugar diets compared to females.

While many of the sex differences in alcohol or HFD consumption involve one sex being more resistant to certain behavioral and physiological changes, in one specific case male and female mice exhibited opposite responses to the effects of HFD on explorative locomotor behaviors in an open field assay. While females had reduced activity in the open field compared to males, exposure to a HFD produced increases to their activity; conversely, males consuming HFD had reductions in open field movement. Interestingly, the current finding that females exhibit increased movement in an open field when consuming HFD has been found previously in the few studies that have used B6 mice in this fashion (Hwang et al., 2010; Krishna et al., 2015). However, studies investigating the effects of HFD on male B6 mice have produced mixed results. While the current study and others (Funkat, Massa, Jovanovska, Proietto, & Andrikopoulos, 2004; Kennedy et al., 2007) show reduced open field movement during HFD treatment, others (Zemdegs et al., 2016; Liu, Zhai, Li, & Ji, 2014; Liu, Zhu, Kalyani, Janik, & Shi, 2014; Heyward et al., 2012) have found no differences, all using male B6 mice. It is also worth noting that female rats fed HFD also seem to increase activity in the open field (Warneke, Klaus, Fink, Langley‐Evans, & Voigt, 2014) while male rats can show either reduced (Sharma, Zhuang, & Gomez‐Pinilla, 2012) or no alteration (Souza et al., 2007) to activity behaviors when consuming HFD. Lastly, many of these aforementioned differences in the open field were found during the first 5 minutes and were no longer present for the second half. These results indicate that in male mice HFD may produce alterations in novelty seeking behavior, as the first 5 min may be viewed as response to a novel environment that wanes over the course of the full 10 min. Indeed, male rodents fed HFD show less novel object exploration than controls and the antidepressant ketamine can restore novelty seeking behaviors (Dutheil, Ota, Wohleb, Rasmussen, & Duman, 2016). In summary, the effects of HFD on open field locomotion may be consistently increased in females, while increases in activity rarely occur in males and more likely either decreases or no changes to activity usually occur regardless of rodent model (rat or mouse). Although HFD did not produce changes in the true anxiety‐like measures for the open field (center time) or light‐dark box in this experiment, a human study investigating depression and anxiety found that HFD increases both disorders in men but not women (Bonnet et al., 2005). Additional studies utilizing both male and female rodent models are needed to further understanding as to why male and female organisms may have opposing behavioral responses to HFD intake.

Even without any of the experimental treatments, home‐cage locomotor activity, as opposed to open field exploratory locomotor behavior, was much greater in female mice than male mice. Of particular note, another study has shown that this difference in activity still can persist even if female rodents are ovariectomized (Chu, Gagnidze, Pfaff, & Ågmo, 2015). Although removal of the ovaries does not affect locomotor activity, centrally located (i.e., brain) estrogens and estrogen receptors (ER) may still be implicated in why female mice exhibit more movement than males. Removal of ERs from the medial preoptic area (a main area in controlling locomotor behavior) leads to the reduction in home‐cage running‐wheel activity as well as open field locomotion, and estrogen replacement restores that activity (Ogawa, Chan, Gustafsson, Korach, & Pfaff, 2003). In addition, sex differences were revealed in activity levels in response to HFD consumption. The current results add to the body of evidence that shows HFD consumption can affect locomotor activity behaviors, as male mice consuming HFD for at least several weeks exhibited reduced locomotor activity compared to controls and as time progressed, as was discovered previously in male mice (Kohsaka et al., 2007). Conversely, female mice had no alterations to locomotor activity, again illustrating that female B6 mice are not affected as much as males by the neurobehavioral effects of HFD consumption. Lastly, while the current study found no effect of alcohol consumption on locomotor activity, a previous study showed reduced bout length and increased counts per bout in ethanol‐consuming male B6 mice (Brager, Ruby, Prosser, & Glass, 2010). These differences are most likely due to ethanol dosing (current study used 10% and the other study 15%) and to the lighting schedule (standard LD cycle vs. skeleton photoperiod).

Both alcohol and HFD are known to produce alterations to BDNF levels in specific parts of the brain. The hippocampus is particularly sensitive to changes in BDNF during alcohol (Darlington, McCarthy, Cox, & Ehringer, 2014) and HFD (Molteni, Barnard, Ying, Roberts, & Gómez‐Pinilla, 2002) exposure but other parts of the brain are sensitive as well including the hypothalamus (Liu, Zhai, et al., 2014; Liu, Zhu, et al., 2014) and prefrontal cortex (Kanoski, Meisel, Mullins, & Davidson, 2007). Although neither HFD nor alcohol led to alterations in whole‐brain BDNF, the current results illustrate male mice exhibit reduced levels of brain‐wide BDNF compared to female mice. As both male and female mice were separately normalized to their respective controls (RC H_2_O), the small reduction in BDNF seen in the male mice is likely due to the diet and alcohol experimental treatments, which would indicate that males may be slightly more susceptible to changes in BDNF with altered diet and ethanol consumption compared to females. There are several other studies that show that sex differences may be present in BDNF levels and in responses to experimental treatments. For example, BDNF levels in the ventromedial hypothalamus (the area which controls feeding behaviors) are reduced in male rats given a HFD but are not altered in females (Liu, Zhai, et al., 2014; Liu, Zhu, et al., 2014). Additionally, the BDNF Val66Met polymorphism, which leads to reductions in proBDNF (the precursor to active BDNF), is a predictor of major depressive disorder in male humans but not in females (Verhagen et al., 2010). Some studies have even shown that BDNF levels are initially higher in female animals (Liu, Zhai, et al., 2014; Liu, Zhu, et al., 2014) and humans (Piccinni et al., 2008) than males. These studies suggest that sex differences in BDNF may play a role in its response to changes in diet and alcohol consumption and that males are more sensitive to changes BDNF than females.

Despite consuming lower doses of ethanol (in terms of g/kg), mice consuming both HFD and alcohol showed improved glucose tolerance compared to animals consuming HFD without ethanol. Both sexes consuming HFD showed moderate improvements to glucose clearance when consuming alcohol. Numerous studies have illustrated that moderate ethanol consumption can improve the symptoms of T2DM (as reviewed by Pietraszek, Gregersen, & Hermansen, 2010). This beneficial effect of moderate ethanol consumption on improving glucose tolerance seems to be similar between males and females (Carlsson et al., 2005). One potential mechanism for this improvement might be due to moderate alcohol consumption improving insulin sensitivity (Joosten, Beulens, Kersten, & Hendriks, 2008), although this effect on insulin sensitivity is more commonly found in human females than in males (Bonnet et al., 2012). As insulin levels or secretion was not altered by ethanol consumption in this experiment, increased insulin sensitivity through alcohol consumption can explain the improved glucose clearance seen in those hyperglycemic and hyperinsulinemic animals, although an insulin sensitivity test was not conducted in this study. Another possibility, related to insulin sensitivity, is that moderate ethanol consumption can promote GLUT4 upregulation, which in turn would increase glucose uptake by striated muscle (Elmadhun, Lassaletta, Burgess, Sabe, & Sellke, 2013). Conversely, increased alcohol concentrations promote downregulation of GLUT4 (Qu et al., 2011) and promote insulin resistance (Lindtner et al., 2013). Future studies can be conducted to determine the mechanism as to why moderate ethanol consumption can lead to improvements to diabetic symptoms while binge drinking exacerbates them.

In conclusion, this study presents results demonstrating sex differences in response to HFD and alcohol consumption for a wide variety of behavioral and physiological assays in B6 mice. Even without any treatments, female mice exhibited increased rearing in the open field, transitions in the light‐dark Box, and increased locomotor activity compared to males. Additionally, female mice had reduced HFD and increased ethanol preferences. Explorative behaviors in the open field and light‐dark Box were reduced in male mice consuming HFD, but increased in females; males were also slightly more sensitive to alterations in BDNF than females. Male mice also exhibited increased sensitivity to the negative physiological consequences of HFD and high‐sugar exposure, showing increased body weight, insulin, and leptin, and reduced glucose tolerance compared to females. Despite drinking significantly more ethanol than males, female mice were either unaffected or similarly affected behaviorally and physiologically compared to males. Lastly, there are some similarities between the two sexes as well. Alcohol consumption increased anxiety levels and improved glucose tolerance in both sexes. Additional studies which investigate sex differences using animal models will be of enormous import as they can provide a foundation and basis for future clinical work.

## Conflict of Interest

The authors declare that they have no conflict of interest.
